# Epidemiology and public health response to a meningitis outbreak in Ghana's Upper West Region, 2025: a descriptive cross-sectional study

**DOI:** 10.11604/pamj.2026.53.80.51188

**Published:** 2026-02-13

**Authors:** Simon Aabalekuu, Ibrahim Mumuni, Justine Bangniyel, Collins Boateng Danquah, Linus Baatiema, Ishmael Kunateh Alhassan, Mark Asare Owusu, Stephen Nimirkpen, Barnabas Bessing

**Affiliations:** 1 Ghana Health Service, Upper West Regional Health Directorate, Wa, Upper West Region, Ghana,; 2 School of Public Health, Faculty of Public Health, University of Port Harcourt, Port Harcourt, Nigeria,; 3 University of Technology and Applied Sciences, Upper East Region, Ghana,; 4 Centre for Migration, Security and International Relations, University of Business and Integrated Development Studies, Wa, Ghana,; 5 Ghana Field Epidemiology and Laboratory Training Programme (GFELTP), School of Public Health, University of Ghana, Accra, Ghana,; 6 Ghana Health Service, Wa Municipal Hospital, Wa, Upper West Region, Ghana,; 7 Department of Veterinary Medicine, Disease Dynamics Unit, University of Cambridge, Cambridge, United Kingdom,; 8 Field Epidemiology and Applied Biostatistics, School of Public Health, Kwame Nkrumah University of Science and Technology (KNUST), Kumasi, Ghana,; 9 The Vaccine-Preventable Diseases (VPD) Unit, World Health Organization (WHO) African Regional Office (AFRO), Harare, Zimbabwe

**Keywords:** *Streptococcus pneumoniae*, *Neisseria meningitidis*, meningitis outbreak, surveillance, Upper West Region, Ghana

## Abstract

Bacterial meningitis remains a recurrent public health threat in the African meningitis belt despite vaccination efforts. Following the introduction of the Meningococcal Africa vaccine, shifts in causative pathogens have been observed. In early 2025, a meningitis outbreak occurred in Ghana´s Upper West Region, necessitating a detailed epidemiological and response assessment. We conducted a retrospective descriptive cross-sectional analysis of surveillance data from all 11 districts of the Upper West Region, Ghana. Records of suspected meningitis cases reported between epidemiological weeks 1 and 15 of 2025 were reviewed. Data were obtained from regional line lists, case-based investigation forms, laboratory reports, and weekly situational updates. Variables analysed included age, sex, district, laboratory confirmation, bacterial aetiology, case fatality rate, and outbreak response activities. A total of 228 suspected meningitis cases and 17 deaths were reported, yielding an overall case fatality rate of 7.7%. Thirty-six cases (15.8%) were laboratory-confirmed, with Streptococcus pneumoniae accounting for 86.1% of confirmed infections, followed by Neisseria meningitidis (5.6%) and Haemophilus influenzae (5.6%). Adolescents and young adults aged 10-29 years were most affected, with a slight male predominance. The outbreak peaked during epidemiological weeks 7-8, with spatial clustering in the Wa Municipal, Nadowli-Kaleo, and Nandom districts. Public health response measures included activating rapid response teams, intensifying surveillance through the surveillance outbreak response management and analysis system and retraining over 250 health workers. Key challenges identified were limited laboratory capacity, inadequate diagnostic tools, and insufficient sustainable funding. The 2025 meningitis outbreak in Ghana´s Upper West Region demonstrates a clear shift towards pneumococcal predominance within the meningitis belt. While coordinated multisectoral response efforts successfully curtailed transmission, persistent gaps in diagnostic capacity, human resources, and sustainable financing threaten future preparedness. Strengthening subnational laboratory systems, expanding multivalent vaccination strategies, and securing predictable outbreak-response funding are critical to achieving the World Health Organization´s defeating meningitis by 2030.

## Introduction

Meningitis is a life-threatening inflammatory condition of the meninges. It remains a major global public health challenge, particularly in low- and middle-income countries in sub-Saharan Africa, where morbidity and mortality are highest [1,2]. Epidemic meningitis is primarily caused by *Neisseria meningitidis, Streptococcus pneumoniae*, and *Haemophilus influenzae*, which continue to drive outbreaks in vulnerable populations [3]. The African meningitis belt, spanning 26 countries from Senegal to Ethiopia, is the world´s most epidemic-prone region for meningitis [4]. Outbreaks are strongly seasonal, occurring predominantly during the dry season, when low humidity, dusty Harmattan winds, and overcrowded living conditions facilitate respiratory transmission [5,6]. Ghana´s Upper West Region (UWR) lies entirely within this belt and has experienced recurrent seasonal outbreaks for decades. Surveillance data show peak transmission between November and April, with approximately 77.6% of cases occurring among individuals under 30 years of age, highlighting persistent age-related vulnerability [7]. Over the past two decades, the epidemiology of bacterial meningitis in the UWR has evolved, marked by shifts in pathogen dominance and persistent seasonality. Between 2009 and 2013, *Streptococcus pneumoniae* (48.2%) and *Neisseria meningitidis* serogroup W135 (40.3%) were the principal causative agents [7]. Subsequent analyses showed sustained spatial clustering in high-risk districts, including Nandom, Jirapa, and Nadowli-Kaleo, with children, adolescents, and young adults aged 5-29 years remaining the most affected groups [8]. More recent investigations have confirmed *Streptococcus pneumoniae* as the dominant pathogen, accounting for 68.37% of cases, followed by *Neisseria meningitidis* serogroup X (27.55%) [9].

Despite changes in pathogen distribution, dry-season seasonality and the concentration of cases among younger populations remain defining epidemiological features. The introduction of the Meningococcal Africa Vaccine (MenAfriVac) led to a dramatic reduction in disease caused by N. meningitidis serogroup A across the African meningitis belt, marking a major public health milestone. Post-introduction surveillance in Burkina Faso documented only six confirmed serogroup A cases between 2011 and 2015 [10]. However, this success has been accompanied by a serogroup replacement phenomenon. Evidence from Ghana indicates a rise in *Neisseria meningitidis* serogroup X and a decline in serogroup W, while other studies show increasing dominance of non-A meningococcal serogroups and *Streptococcus pneumoniae* [11,12]. These trends underscore the need for continuous surveillance and adaptive immunisation strategies. In early 2025, a meningitis outbreak occurred in Ghana´s UWR, providing an opportunity to assess the current epidemiology of meningitis in a high-risk setting. The region remains vulnerable due to ecological conditions, cross-border population movement, and constrained diagnostic capacity, which sustain recurrent epidemics despite ongoing surveillance and vaccination efforts. This study analyses cases reported during the first 15 epidemiological weeks of 2025, examining demographic and clinical characteristics, laboratory-confirmed pathogens, case fatality rates, and spatial transmission patterns. By integrating surveillance and laboratory data, the study evaluates the performance of surveillance, case management, and laboratory systems under outbreak conditions and identifies operational gaps relevant to strengthening subnational preparedness and supporting the World Health Organization´s “Defeating Meningitis by 2030” roadmap.

## Methods

This descriptive cross-sectional study adheres to the Strengthening the Reporting of Observational Studies in Epidemiology (STROBE) guidelines for transparent reporting [13].

**Study design:** this study was a descriptive cross-sectional analysis of surveillance data from the 2025 meningitis outbreak in the UWR of Ghana. The investigation focused on summarising the epidemiological characteristics of reported cases, including demographic, clinical, and laboratory variables. Data were extracted from the regional meningitis line list maintained by the Ghana Health Service, with verification from case-based investigation forms and laboratory result records where necessary.

**Figure 1:**
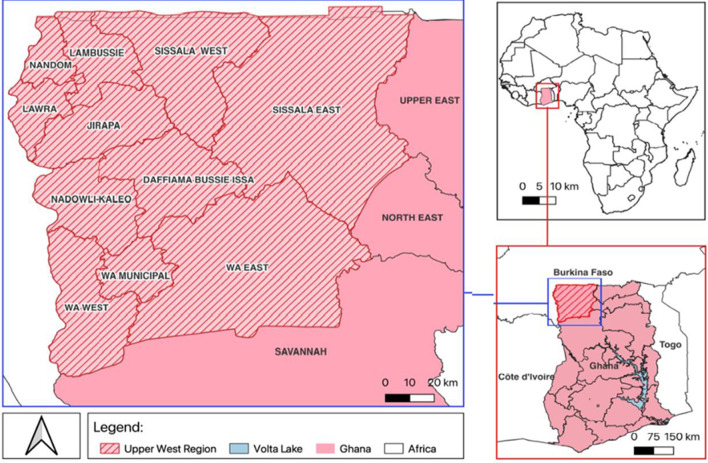
map of the Upper West Region

Study area and setting: the study was conducted in the UWR of Ghana (Figure 1), located in the north-western part of the country. The region is bordered by the Savannah Region to the south, the Upper East and North-East Regions to the east, and the Republic of Burkina Faso to the north and west. It lies between latitudes 9°35’N and 11°00’N and longitudes 1°25’W and 2°50’W, covering a land area of 18,476 km^2^ [14]. Administratively, the UWR comprises 11 districts: Daffiama-Bussie-Issa, Jirapa, Lambussie-Karni, Lawra, Nadowli-Kaleo, Nandom, Sissala East, Sissala West, Wa East, Wa Municipal, and Wa West, with Wa Municipal serving as the regional capital. The population increased from 702,110 in 2010 to 901,502 in 2020, of which 73.6% reside in rural areas [15,16]. The UWR lies within the African meningitis belt and is characterised by a tropical climate with a single rainy season from May to October and a prolonged dry Harmattan season from November to April. Average temperatures range from 22.6 °C to 40.0 °C, and relative humidity during the dry season may fall to approximately 16%, creating conditions conducive to meningitis transmission [8,9]. Health services are delivered through a network of 488 public and private health facilities [14]. The Upper West Regional Health Directorate provides policy coordination, technical oversight, and supervisory support to district, sub-district, and community-level health services across the region.

**Study population:** the study population consisted of records of all suspected meningitis cases who presented to health facilities in the UWR for medical care during the 2025 outbreak period. These cases were captured in the regional meningitis line list, with additional information obtained from case-based investigation and laboratory reporting forms.

### Case and outbreak definitions used in data abstraction

**Suspected meningitis:** a case of suspected meningitis was defined as any person with a sudden onset of fever (>38.5°C rectal or 38.0°C axillary) and one of the following signs: neck stiffness, flaccid neck (infants), bulging fontanelle (infants), convulsion, or other meningeal signs.

**Confirmed meningitis:** a confirmed meningitis case was defined as any person presenting with clinical signs of meningitis and with laboratory confirmation of a causative pathogen, *Neisseria meningitidis* (*N. meningitidis*), *Streptococcus pneumoniae* (Spn), or *Haemophilus influenzae* type b (Hib), isolated from cerebrospinal fluid (CSF) using polymerase chain reaction (PCR).

**Alert threshold:** the alert threshold was defined as an attack rate of 3 suspected cases per 100,000 inhabitants per week in a district or subdistrict with a population between 30,000 and 100,000; or as 2 cases in 1 week, or a higher incidence than in a non-epidemic year (in populations < 30,000). Crossing this threshold triggers the reinforcement of surveillance.

**Epidemic threshold:** the epidemic threshold was defined as an attack rate of 10 cases per 100,000 inhabitants in 1 week in a district or sub-district, or 10 per 100,000 if considered at high risk of an epidemic (in populations ≥30,000); or as 5 cases in 1 week, or a doubling of incidence in a 3-week period (in populations <30,000). Crossing this threshold triggers the launch of vaccination campaigns when the predominance of *Neisseria meningitidis* is confirmed, and the use of a specific antibiotic treatment protocol.

**Data collection and data analysis:** data were collected from weekly meningitis situational reports and the regional line list compiled by the Disease Surveillance Unit of the Upper West Regional Health Directorate. The dataset included demographic, clinical, laboratory, and geographic information for all reported meningitis cases during the outbreak period. Additional verification was conducted using case-based investigation forms and laboratory result reports, as necessary, to ensure data completeness and accuracy. For analysis, frequencies and proportions were calculated for categorical variables, including sex, age group, and district of residence. An epidemic curve (epi-curve) was generated from the line list to illustrate the temporal trend of cases. District-specific case fatality rates (CFRs) were calculated to assess geographical variations in mortality, and the distribution of isolated bacterial strains was examined to identify the predominant etiologic agents. Results were summarised and presented in tables, graphs, and maps for ease of interpretation. All analyses were performed using Microsoft Excel 2010.

**Ethical consideration:** this study was conducted as part of the public health response to the 2025 meningitis outbreak in the UWR of Ghana. The analysis utilised routine surveillance data collected under the national Integrated Disease Surveillance and Response (IDSR) system. As the investigation was part of an emergency public health activity, formal ethical approval was not required according to the Ghana Health Service policy. No personal identifiers were included in the dataset, and all data were handled with strict confidentiality to ensure anonymity and privacy. Permission to use the data was obtained from the Upper West Regional Health Directorate.

## Results

**Epidemiological overview:** between January and mid-April 2025 (epidemiological weeks 1-15), a total of 228 suspected meningitis cases and 17 deaths were reported in the UWR of Ghana. Case notifications increased steadily from the early weeks, peaking between weeks 7 and 8, before declining sharply following the implementation of intensified surveillance and control measures. No confirmed cases were detected during the final four weeks, and no deaths occurred in the last six weeks of the reporting period, indicating that transmission had been effectively controlled.

**Laboratory findings:** of the 228 suspected cases, 36 (15.8%) were confirmed by laboratory testing. *Streptococcus pneumoniae* was identified as the predominant pathogen, accounting for 31 cases (86.1%), followed by *Neisseria meningitidis* (5.6%) and *Haemophilus influenzae* (5.6%). One case involved co-infection with *Streptococcus pneumoniae* and *N. meningitidis*. Confirmatory testing, utilising Gram staining and PCR, enabled the timely identification of pathogens to inform case management and guide outbreak response actions.

**Figure 2:**
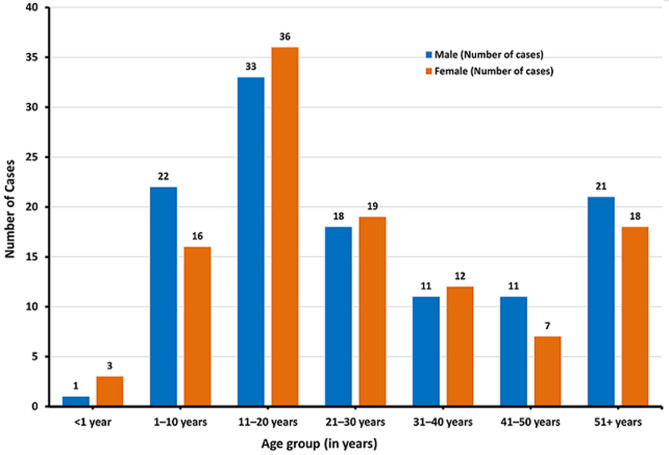
age-sex distribution of suspected meningitis cases in 2025, Upper West Region

**Age and sex distribution of suspected meningitis cases:** analysis of age and sex distribution (Figure 2) shows that meningitis predominantly affected adolescents and young adults. The 11-20-year age group recorded the highest number of suspected cases, followed by the 21-30-year and 51+ year groups. Both sexes were affected, though males slightly outnumbered females overall, particularly in the 11-20-year age band. This pattern suggests higher exposure among school-aged and working-age males, possibly linked to outdoor activities, occupational risk, and social mixing patterns. The distribution underscores the need for targeted health education and preventive interventions among adolescents and young adults, who remain a key transmission group during meningitis outbreaks.

**Figure 3:**
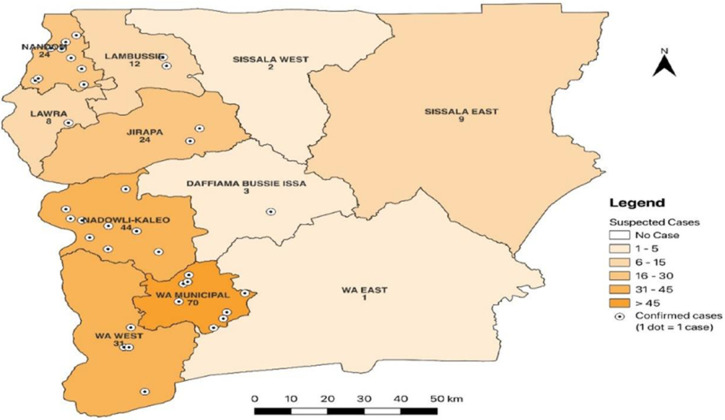
spot map of suspected and confirmed cases, Upper West Region, Week 1 - Week 15, 2025

**Geographical trend:** meningitis cases were reported across all 11 districts of the UWR, demonstrating clear geographic and temporal variation in both caseload and outcomes (Table 1). The number of suspected cases increased steadily from January, peaking around epidemiological weeks 7-8, before declining after the implementation of intensified control measures. The highest case burden was observed in Wa Municipal (n = 70), followed by Nadowli-Kaleo (n = 44) and Wa West (n = 31). In total, 36 cases (15.8%) were laboratory confirmed and 17 deaths were recorded, corresponding to an overall case fatality rate (CFR) of 7.7%. The highest CFRs were reported in Nandom (20.0%), Lawra (12.5%), and Nadowli-Kaleo (11.9%), while Sissala East, Sissala West, and Wa East recorded no confirmed cases or deaths. These findings reveal marked subregional disparities, with the central and northwestern districts bearing the heaviest disease burden and mortality. The spatial distribution of suspected and confirmed meningitis cases across the UWR shows a heterogeneous pattern, with clusters concentrated in Wa Municipal, Nadowli-Kaleo, and Wa West districts (Figure 3). These areas reported the highest case counts (>30 cases), corresponding to the most intense transmission zones. Several northern districts, including Nandom, Lawra, and Lambussie, also recorded moderate case numbers, while Sissala East, Sissala West, and Wa East reported few or no cases. The central and northwestern corridor emerged as the principal hotspot of the outbreak, reflecting likely differences in population density, environmental conditions, and healthcare access. The distribution further highlights the importance of district-specific surveillance and response strategies to contain future epidemics.

**Table 1:** distribution of meningitis cases by districts/municipals, Upper West Region, 2025

District	Cases	Confirmed	Deaths	Case fatality rate (CFR)
Daffiama-Bussie-Issa	3	1	0	0.0
Jirapa Municipal	24	2	1	4.3
Lambussie	12	2	1	9.1
Lawra Municipal	8	1	1	12.5
Nadowli/Kaleo	44	9	5	11.9
Nandom Municipal	24	9	5	20.0
Sissala East Municipal	9	0	0	0.0
Sissala West	2	0	0	0.0
Wa East	1	0	0	0.0
Wa Municipal	70	8	2	3.0
Wa West	31	4	2	6.5
**Total**	228	36	17	7.7

UWR**:** Upper West Region

**Figure 4:**
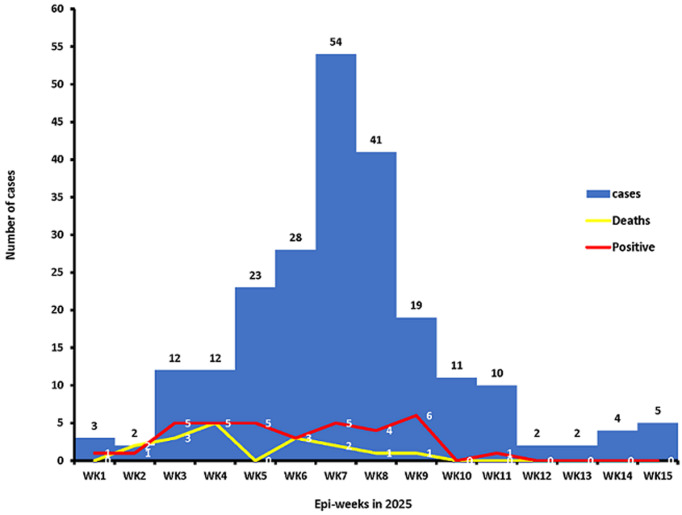
epi-curve of meningitis outbreak in the Upper West Region, Week 1 - Week 15, 2025

**Epidemic curve:** the epidemic curve (Figure 4) depicts a gradual rise in suspected meningitis cases beginning in Week 2, reaching a sharp peak between Weeks 7 and 8, followed by a steady decline through Week 15. The red line shows laboratory-confirmed cases (positives), while the yellow line represents deaths. The temporal pattern indicates an acute, time-limited outbreak consistent with meningitis seasonality in the northern belt of Ghana. The peak coincided with the dry Harmattan period, suggesting that environmental factors influenced transmission. The decline after Week 8 corresponds with intensified public-health interventions, including improved case management, enhanced surveillance, and community engagement, ultimately leading to zero deaths in the final six weeks.

**Figure 5:**
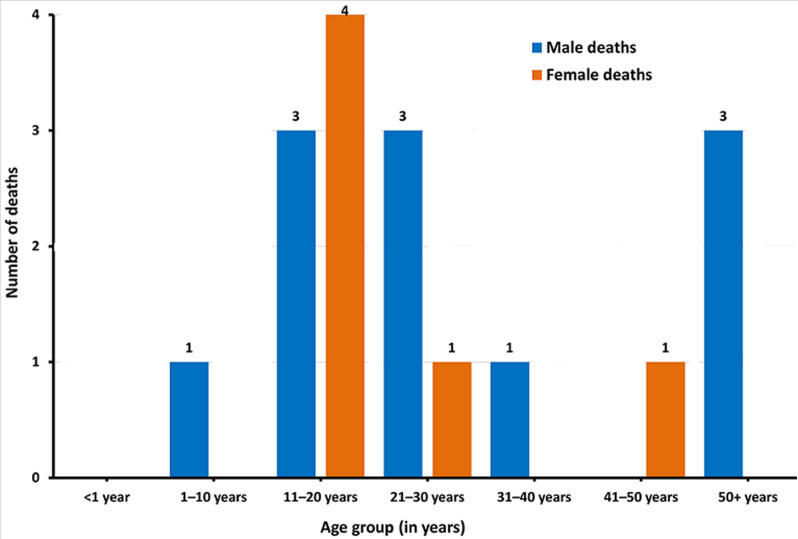
age-sex distribution of meningitis deaths in 2025, Upper West Region

**Figure 6:**
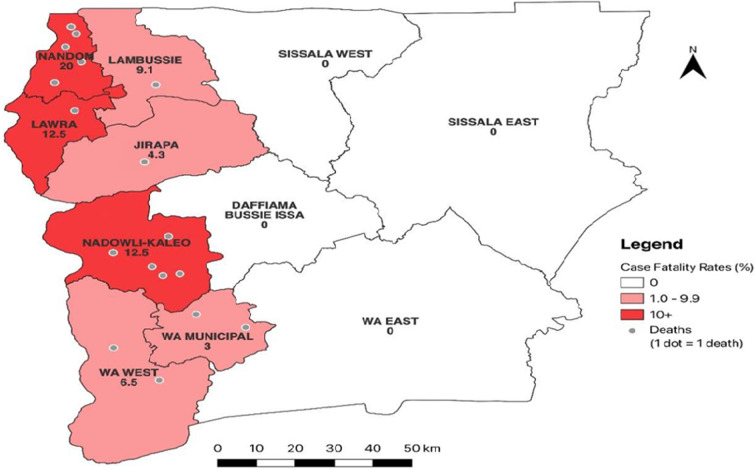
case fatality rate and deaths by districts, Upper West Region, Week 1 - Week 15, 2025

**Mortality patterns by age, sex, and district:** mortality was highest among adolescents and young adults, reflecting the overall case distribution (Figure 5, Figure 6). The 11-20-year age group accounted for the most significant proportion of deaths, followed by those aged 21-30 years and 50 years and above. Males experienced marginally more deaths than females across most age categories, indicating higher exposure and possibly delayed care-seeking among men. The increased case fatality observed in younger and older populations may be due to late presentation to health facilities, misdiagnosis at peripheral levels, or limited access to advanced clinical care. These trends underscore the need for improved case management protocols, early referral, and aggressive treatment, particularly for adolescents and older adults in rural settings where access to care is limited. Spatially (Figure 6), case fatality rates (CFRs) varied significantly across districts. Nandom Municipal (20.0%), Lawra (12.5%), and Nadowli-Kaleo (11.9%) reported the highest CFRs, despite having fewer cases, suggesting possible gaps in clinical management and delayed referrals. Conversely, Wa Municipal, which recorded the highest number of cases (n = 70), reported a relatively low CFR of 3.0%, probably due to better healthcare access and case management capacity. These findings emphasise the importance of strengthening early detection, referral pathways, and clinical care, particularly in rural and resource-limited districts, to reduce mortality in future outbreaks.

**Summary of key epidemiological patterns:** overall, the 2025 outbreak mainly affected adolescents and young adults (10-29 years), with males being more frequently impacted, and notable spatial clustering was observed in historical hotspot districts. Although the outbreak was finally contained through coordinated response efforts, the findings emphasise persistent weaknesses in diagnostic capacity and subnational health system readiness that need targeted strengthening before future epidemics.

### Public health response

**Surveillance and coordination:** a multi-layered surveillance and coordination mechanism was rapidly activated to guide the outbreak response, enabling timely case detection, data sharing, and coordinated decision-making across all levels of the health system. Weekly coordination meetings were held at district and regional levels, bringing together the Ghana Health Service (GHS), World Health Organization (WHO), local authorities, and partners to review surveillance data, identify gaps, and align response actions. The Surveillance Outbreak Response Management and Analysis System (SORMAS) was used for real-time case notification, tracking, and analysis. Interactive dashboards supported early identification of transmission hotspots and informed evidence-based response decisions. Action points from coordination meetings were monitored by designated focal persons to ensure accountability and follow-up. Multidisciplinary Rapid Response Teams (RRTs), comprising epidemiologists, clinicians, laboratory scientists, and risk communication specialists, were deployed to affected districts. The teams conducted active case searches, verified surveillance data, supported sample collection and case management, and provided on-site mentorship and refresher training for frontline health workers. Standardised case definitions for suspected, probable, and confirmed meningitis were harmonised with WHO and GHS guidelines and disseminated to all health facilities and community surveillance structures. Community-based surveillance (CBS) was strengthened by retraining and engaging community-based surveillance volunteers (CBSVs) to identify early symptoms and promptly report suspected cases. Community-based surveillance volunteers were equipped with standardised tools and supported by traditional, religious, and community leaders to promote trust, counter rumours, and encourage early health-seeking behaviour, particularly in hard-to-reach communities. The regional Emergency Operations Centre (EOC) was activated to coordinate logistics, data management, and partner support. The EOC facilitated daily situation updates, integrated reporting, and implementation of a unified incident action plan to guide field operations and resource mobilisation.

**Case management:** it focused on early detection, prompt treatment, and adherence to national and WHO guidelines. A total of 250 health workers were trained on meningitis case management, including clinical recognition, antibiotic therapy, lumbar puncture, and infection prevention and control. Rapid response teams clinicians provided supportive supervision and mentorship at peripheral facilities. Referral pathways were strengthened, and essential antibiotics and consumables were redistributed to high-burden districts. Routine case reviews involving clinical, laboratory, and surveillance teams improved case classification and treatment outcomes. Although mortality declined during the response, gaps in intensive care capacity and staffing at peripheral facilities persisted.

**Laboratory support:** laboratory services played a central role in confirming cases and guiding clinical management. Despite staffing constraints, diagnostic services were sustained through mentorship from RRT laboratory specialists and support from regional reference laboratories. Cerebrospinal fluid (CSF) samples were analysed using Gram stain, latex agglutination, culture, and polymerase chain reaction (PCR), confirming *Streptococcus pneumoniae* as the predominant pathogen. On-site coaching and remote supervision supported adherence to biosafety and quality standards. However, the lack of urine antigen tests for S. pneumoniae limited confirmation in patients unable to undergo lumbar puncture, highlighting a critical diagnostic gap.

**Logistics, data management, and risk communication:** prompt procurement and partner support ensured the availability of essential supplies, including CSF collection kits, reagents, culture media, and antibiotics. Transport media and cold boxes facilitated safe specimen transfer to regional and national reference laboratories, including the Zonal Public Health Reference Laboratory. Laboratory and surveillance data were integrated through SORMAS, enabling real-time linkage of clinical, laboratory, and epidemiological information. Daily data reviews at district and regional levels guided response prioritisation and resource allocation, while continuous data validation improved reporting quality. Risk communication and community engagement were integral to the response. Daily radio programmes, broadcast in local languages, provided information on symptoms, transmission, and prevention. Community outreach activities in markets, schools, and places of worship promoted early care-seeking and preventive behaviours. Information, Education, and Communication (IEC) materials reinforced messages, while rumour-tracking mechanisms enabled rapid identification and correction of misinformation.

**Challenges:** despite a timely and coordinated response, several challenges constrained effectiveness and sustainability. Inadequate and short-term financing limited continuity of surveillance, community engagement, and post-outbreak follow-up once emergency funds were depleted. Shortages of trained laboratory personnel at the district level delayed case confirmation and increased reliance on reference laboratories. Inadequate diagnostic capacity, particularly the absence of rapid, non-invasive tests for *Streptococcus pneumoniae*, restricted confirmation of cases and constrained antibiotic stewardship. Addressing these gaps is essential for improving preparedness and response to future meningitis outbreaks.

## Discussion

This study describes the 2025 meningitis outbreak in Ghana´s Upper UWR and reaffirms the region´s persistent vulnerability within the African meningitis belt. A total of 228 suspected cases and 17 deaths were reported, corresponding to a CFR of 7.7%, with *Streptococcus pneumoniae* accounting for the majority of laboratory-confirmed infections. The outbreak predominantly affected adolescents and young adults and exhibited clear spatial clustering in Wa Municipal, Nadowli-Kaleo, and Nandom districts. Transmission peaked between epidemiological weeks 7 and 8 and declined following intensified surveillance, rapid response deployment, and enhanced community engagement. Despite these measures, gaps in diagnostic capacity, shortages of trained personnel, and limited sustainable funding constrained the effectiveness of outbreak containment, underscoring the need for strengthened preparedness and laboratory systems. The epidemiological pattern observed in this outbreak reflects the broader and evolving challenges of meningitis control across the African meningitis belt, a region characterised by ecological vulnerability, seasonal climatic extremes, and constrained access to healthcare [8,9]. The dominance of *Streptococcus pneumoniae* (86.1%) is consistent with post-MenAfriVac epidemiological shifts observed across West Africa. While the MenAfriVac vaccine has led to a dramatic reduction in *Neisseria meningitidis* serogroup A disease since its introduction in 2010, this success has been accompanied by serogroup and pathogen replacement, with increasing contributions from non-A meningococcal serogroups (including W and C) and *Streptococcus pneumoniae* [3,8,9,11,12]. The findings from this outbreak further support calls for expanded multivalent conjugate vaccination strategies that address the current pathogen landscape [2].

The age distribution of cases, with approximately one-third occurring among individuals aged 11-20 years, aligns with previous studies identifying adolescents and young adults as high-risk groups for meningitis in northern Ghana [7,8]. Increased social mixing, school attendance, crowded living conditions, and gaps in immunisation coverage may contribute to this vulnerability. These findings highlight the importance of age-targeted prevention strategies, including adolescent-focused vaccination approaches and school-based risk communication, as integral components of meningitis control in high-risk settings. Temporally, confirmed cases occurred between epidemiological weeks 1 and 8, coinciding with the dry Harmattan season, which spans November to April. This period is characterised by high temperatures, dry, dusty winds, and low humidity, which facilitate nasopharyngeal colonisation and transmission of respiratory pathogens such as *Streptococcus pneumoniae* [7-9]. The seasonal pattern observed in this outbreak is consistent with historical trends in the UWR and reinforces the need for heightened surveillance and preparedness ahead of the dry season [7,8]. Spatial clustering in Wa Municipal, Nadowli-Kaleo, and Nandom districts mirrors patterns documented in earlier outbreaks, including analyses from 2018 to 2020 that identified these districts as recurrent hotspots [8]. The persistence of these geographic patterns likely reflects a combination of population density, mobility, environmental exposure, healthcare access, and variability in diagnostic and reporting capacity. These findings emphasise the value of geographically targeted interventions, such as strengthening district-level laboratory networks, deploying mobile response teams, and tailoring risk communication strategies to local socio-cultural contexts. The CFR observed in this outbreak (7.7%) is lower than the 12.2% reported between 2009 and 2013 in the same region [7], suggesting incremental improvements in outbreak detection and case management. However, it remains above the WHO-recommended threshold of less than 5% for well-managed meningitis outbreaks [2]. Persistently elevated mortality likely reflects delayed care-seeking, transportation barriers, late presentation to health facilities, and limitations in rapid diagnostics and advanced case management, particularly in rural and underserved districts. Strengthening early case detection, improving referral pathways, and ensuring timely access to appropriate antibiotics remain critical to reducing meningitis-related mortality.

**Study strengths and limitations:** the 2025 meningitis outbreak in Ghana´s UWR highlighted both the strengths and limitations of the regional health system. The response was swift and well-coordinated, featuring enhanced surveillance through SORMAS, rapid deployment of multidisciplinary rapid response teams (RRTs), retraining of over 250 health workers, and an intensive multi-channel risk communication campaign using radio, faith-based networks, and community influencers. These efforts facilitated early detection, improved case management, and likely contributed to the rapid containment of the outbreak and reduction in mortality. However, several systemic gaps limited the speed and sustainability of the response. Financial constraints curtailed community surveillance and outreach once emergency funds were depleted. A shortage of trained laboratory personnel delayed CSF analysis, and the absence of urine antigen testing for *Streptococcus pneumoniae* reduced diagnostic capacity. Reliance on secondary surveillance data may have introduced underreporting and incomplete records, particularly from hard-to-reach areas. Despite these limitations, the study´s integration of epidemiological, laboratory, and spatial analyses using real-time data provides valuable insights to inform future preparedness, diagnostic strengthening, and rapid response mechanisms for meningitis and other epidemic-prone diseases in Ghana and across the African meningitis belt.

**Implications for policy, practice and research:** the outbreak underscores the need for sustained domestic and partner financing to strengthen district-level laboratory infrastructure, expand access to decentralised diagnostic services, and ensure availability of essential meningitis diagnostics. National immunisation policies should adapt to the evolving epidemiology by prioritising the introduction and scale-up of multivalent conjugate vaccines covering additional meningococcal serogroups and *Streptococcus pneumoniae*, in alignment with the WHO Defeating Meningitis by 2030 roadmap. Routine integration of spatial analysis into meningitis surveillance systems is critical for early identification of high-risk districts and timely targeting of response activities. Continuous training and mentorship of frontline health workers, alongside strengthened referral and case-management pathways, are essential to reduce diagnostic delays and mortality. Sustained risk communication and community engagement should remain central to outbreak response, promoting early health-seeking behaviour, countering misinformation, and maintaining public trust. Future research should prioritise molecular and serotype surveillance to better characterise circulating pathogens and guide vaccine policy. Operational research evaluating decentralised diagnostic models, community-based surveillance effectiveness, and cost-effective preparedness financing mechanisms is needed to inform scalable and sustainable meningitis control strategies in high-risk settings.

## Conclusion

The 2025 meningitis outbreak in Ghana’s UWR underscores the persistent vulnerability of populations within the African meningitis belt. The predominance of *Streptococcus pneumoniae*, the concentration of cases among adolescents and young adults, and the high fatality rates in resource-limited districts highlight ongoing gaps in surveillance sensitivity, laboratory capacity, and clinical case management. Nevertheless, the successful containment of the outbreak following coordinated multisectoral action illustrates the value of early detection, rapid laboratory confirmation, and effective community engagement. Going forward, sustained investment in surveillance systems, laboratory infrastructure, health worker training, and community partnerships will be crucial to mitigating the impact of future outbreaks. Strengthening sustainable financing mechanisms and expanding preventive vaccination strategies across high-risk areas will further enhance preparedness and resilience against meningitis and other epidemic-prone diseases in Ghana and throughout the wider African meningitis belt.
